# Euchromatin islands in large heterochromatin domains are enriched for CTCF binding and differentially DNA-methylated regions

**DOI:** 10.1186/1471-2164-13-566

**Published:** 2012-10-26

**Authors:** Bo Wen, Hao Wu, Yuin-Han Loh, Eirikur Briem, George Q Daley, Andrew P Feinberg

**Affiliations:** 1 Center for Epigenetics and Department of Medicine, Johns Hopkins University School of Medicine, Baltimore, MD, USA; 2 Department of Biostatistics and Bioinformatics, Rollins School of Public Health, Emory University, Atlanta, GA, USA; 3 Division of Pediatric Hematology/Oncology, Children's Hospital Boston and Howard Hughes Medical Institute, Boston, MA, USA; 4 Current address: Institutes of Biomedical Sciences, Shanghai Medical College, Fudan University, Shanghai, China

**Keywords:** Epigenetics, H3K9me2, Euchromatin islands, CTCF, DNA methylation

## Abstract

**Background:**

The organization of higher order chromatin is an emerging epigenetic mechanism for understanding development and disease. We and others have previously observed dynamic changes during differentiation and oncogenesis in large heterochromatin domains such as Large Organized Chromatin K (lysine) modifications (LOCKs), of histone H3 lysine-9 dimethylation (H3K9me2) or other repressive histone posttranslational modifications. The microstructure of these regions has not previously been explored.

**Results:**

We analyzed the genome-wide distribution of H3K9me2 in two human pluripotent stem cell lines and three differentiated cells lines. We identified > 2,500 small regions with very low H3K9me2 signals in the body of LOCKs, which were termed as euchromatin islands (EIs). EIs are 6.5-fold enriched for DNase I Hypersensitive Sites and 8-fold enriched for the binding of CTCF, the major organizer of higher-order chromatin. Furthermore, EIs are 2–6 fold enriched for differentially DNA-methylated regions associated with tissue types (T-DMRs), reprogramming (R-DMRs) and cancer (C-DMRs). Gene ontology (GO) analysis suggests that EI-associated genes are functionally related to organ system development, cell adhesion and cell differentiation.

**Conclusions:**

We identify the existence of EIs as a finer layer of epigenomic architecture within large heterochromatin domains. Their enrichment for CTCF sites and DNAse hypersensitive sites, as well as association with DMRs, suggest that EIs play an important role in normal epigenomic architecture and its disruption in disease.

## Background

Epigenetics involves information retained during cell division other than DNA sequence per se, and both DNA methylation and post-translational modifications of histones are fundamental in understanding normal development and disease [1-3]. Genome-scale localization of histone modifications had been extensively mapped in mammalian genomes [4-10]. While most of these studies focused on local regulatory elements such as promoters and enhancers, global organization of the chromatin has not been well understood.

Recent evidence indicates that repressive histone modifications form large scale domains in both mouse and human genomes. We had previously identified large blocks of H3 lysine 9 dimethylation (H3K9me2), termed Large Organized Chromatin K9-modifications (LOCKs), which affect more than 40% of the mouse genome in liver cells [11]. LOCKs significantly overlap with lamina-associated domains (LADs) [12] and are associated with domain-wide gene silencing in a tissue-specific manner. Importantly, both coverage and domain size of LOCKs increase upon differentiation of mouse embryonic stem cells (ESCs) [11]. On the other hand, genome-scale reduction of LOCKs was seen in epithelial-to-mesenchymal transition (EMT) induced by TGF-β treatment of mouse hepatocytes, a process in which cells gain stem cell-like and malignant-type traits [13]. Similarly, large blocks of other repressive marks (H3K9me3 and H3K27me3) are also found to expand in human lung fibroblasts compared with human ESCs [14], and those blocks/LOCKs expand in breast cancer cells relative to normal epithelial cells [15]. Furthermore, large H3K9me3 and H4K20me3 blocks specifically coat olfactory receptor (OR) gene clusters in mouse olfactory epithelium but not in liver [16]. Taken together, these data demonstrate that large heterochromatin domains are highly dynamic in differentiation and tumorigenesis.

DNA methylation has been tightly linked to development and disease [1]. We previously reported that differentially methylated regions (DMRs) related to tissue specificity (T-DMRs), colon cancer (C-DMRs) and reprogramming (R-DMRs) have largely common targets in the genome and are strongly associated with local regulation of adjacent genes [17,18]. Whole genome bisulfite sequencing had found partial methylated domains (PMDs) which are highly methylated in human ESCs but partially methylated in fibroblasts [19]. Similar large hypomethylation blocks relative to normal cells have been identified in colon cancer [20] and breast cancer cells [15], and loss of methylation in these regions is accompanied by acquisition of large domains of H3K9me3 and H3K27me3 [15].

Surprisingly, the relationship of H3K9me2 LOCKs/blocks to DMRs has not been previously assessed. In the course of this investigation, we identified a new chromatin unit we term “euchromatin island” which may serve as a fulcrum between DNA methylation and chromatin in development.

## Results

We analyzed whole genome distribution of H3K9me2 by ChIP-chip using a highly specific monoclonal antibody in two human pluripotent stem cell (PSC) lines (human ESC H1, human iPSC ADA-38) and three primary differentiated cell lines: human astrocytes (HA), human aortic endothelial cells (HAEC) and human pulmonary fibroblasts (HPF). For differentiated cells, we used early passages of primary cells instead of immortalized cell lines to avoid potentially aberrant epigenetic changes due to long time cell culture and immortalization of the cells [21]. These differentiated lines represent three germ layers: ectoderm (HA), mesoderm (HAEC) and endoderm (HPF).

**Figure 1:**
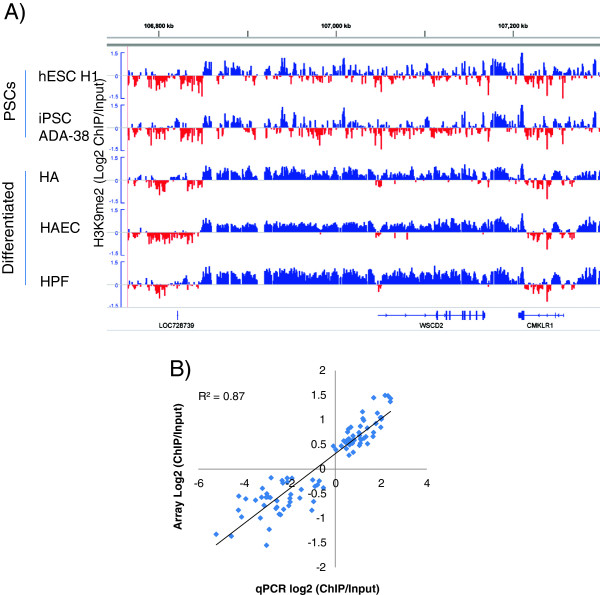
**Global pattern of H3K9me2 in human pluripotent and differentiated cells.A**) A representative region that shows LOCKs in differentiated cells (HA, HAEC and HPF) but not PSCs (H1 and ADA-38). Shown are H3K9me2 signals of ChIP-chip experiments in a ~500 kb long region containing *WSCD2* gene. Positive and negative log2 (ChIP/Input) rations are shown in blue and red, respectively. **B**) qPCR validation of ChIP-chip data. By comparing four cell lines in 23 regions (Figure S1), enrichments (log2 ChIP/Input) measured by ChIP-chip (Y axis) and ChIP-qPCR (X axis) are highly correlated (R^2^ = 0.87).

We normalized the ChIP-chip data as described [11] to calculate the log2 ratios of ChIP/Input comparable among cell types. By using the 90^th^ quantile as a cutoff to define large domains, the genome coverage of LOCKs was found to increase from 17.5-24% in PSC lines, to 39.3-44.8% in differentiated cells, and the average sizes of LOCK expanded from 142–171 kb in PSC lines, to 233–315 kb in the differentiated. The trends were the same when we used different cutoffs to define LOCKs (Additional file 1: Table S1), consistent with our previous findings that LOCKs increase after mouse ESC differentiation [11]. For example, in the *WSCD2* gene locus, only some small H3K9me2 peaks can be seen in the PSCs, but the H3K9me2 enriched regions expanded to ~350 kb long and cover the whole gene body and its flanking regions in the differentiated cells (Figure 1A).

To validate the ChIP-chip data, we performed quantitative PCR (qPCR) on 23 loci using independently prepared ChIP and input DNA samples from four cell types. For all the cases, the quantitative differences of H3K9me2 enrichments within and among samples detected by ChIP-chip were well validated by qPCR (Additional file 2: Figure S1). Overall, the ChIP/Input log2 ratios of microarray (ChIP-chip) and qPCR were strongly correlated (R^2^ = 0.87, Figure 1B), indicating that the ChIP-chip data are of high quality.

To reveal the relationship between dynamics of H3K9me2 and DNA methylation on a large scale, we compared genome-wide distributions of LOCKs (this study), PMDs in fibroblasts [14], and DNA hypomethylation blocks in colon cancer [20]. LOCKs in fibroblasts (HPF) largely overlap PMDs (Additional file 3: Figure S2A), and overall 61.5% regions of LOCKs in HPF coincide with PMDs (p < 0.001, based on 1,000 permutations), and H3K9me2 signals in the regions of PMDs are higher than non-PMD regions (Additional file 3: Figure S2B). Furthermore, more than 80% LOCK regions in HPF were contained within DNA hypomethylation blocks found in colon cancer tissues (Additional file 3: Figure S2). Thus, our data support a strong correlation between LOCKs and DNA hypomethylation blocks in human cells.

**Figure 2:**
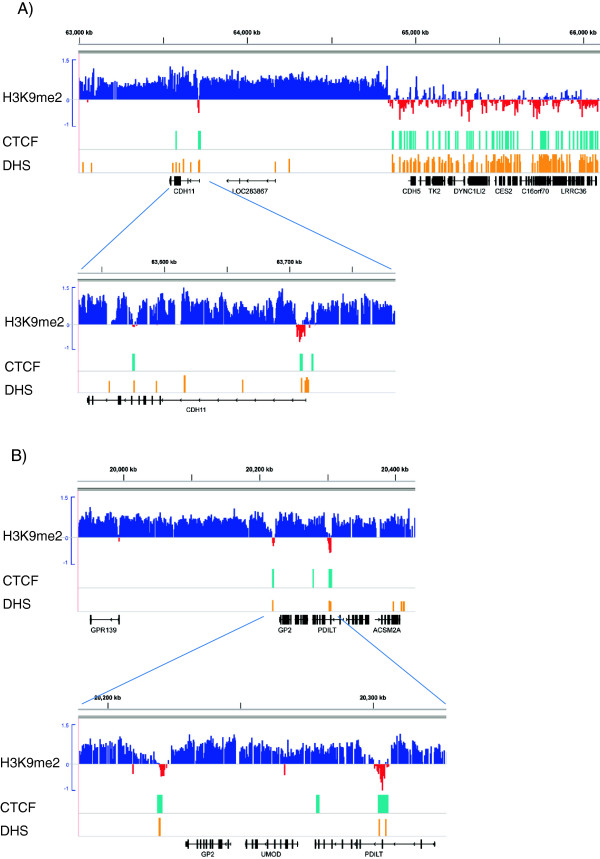
**Euchromatin islands (EIs) in LOCKs overlap CTCF interacting regions and DNase hypersensitive sites (DHSs).** H3K9me2 log ratios of HAEC are shown on the top track. CTCF binding regions and DHSs of HUVEC are denoted as light blue and orange bars, respectively. EIs are small regions with strong negative signals within the body of LOCKs. **A**) Shown is a 3 Mb long region (top) containing CHD11 genes (zoomed-in view on the bottom), a member of the cadherin gene family. CFCF interacting regions and DHSs are highly depleted in the H3K9me2 blocks (LOCKS), but overlap the EI located near the TSS of *CDH11* gene. **B**) Additional examples of EIs near GP2 and PDILT genes.

On closer examination of the microstructure of the LOCKs, we noticed that many small H3K9me2-depleted regions are located in the body of LOCKs. These regions are a few kb in length, and away from the LOCK boundaries. We found that these regions are abundant in the genome, and they appear to be associated with open chromatin (see below). Thus, we termed these regions Euchromatin Islands (EIs). As an example, an EI was found near the transcription start sites (TSSs) of the cadherin 11 gene (*CDH11*, Figure 2A), of which epigenetic disruption was associated with metastasis of human cancers [22]. Other examples of EIs include within the gene body of *PDILT*, a testis-specific gene; and downstream of the glycoprotein 2 (*GP2*) gene (Figure 2B).

Then we developed a statistical algorithm to identify EIs genome-wide (see Methods). We identified 758 to 2,465 EIs across cell types, with average sizes from 4.4 to 5.9 kb (Table 1 and Additional file 4: Table S2). These EIs form strong dips relative to adjacent LOCK regions as demonstrated by average H3K9me2 densities (Additional file 5: Figure S3). We have performed replicates on one array of the “Mouse ChIP-chip 2.1M Whole-Genome Tiling sets”, which covers 10% of the genome. The EIs detected from the replicate experiments have high concordance with the ones from whole genome arrays (Additional file 6: Figure S4). Percentage of EIs detected from whole genome arrays that can also be detected from replicate arrays are 76.3% for H1, 74.1% for ADA-38, 63.3% for HA, 84.4% for HAEC and 71.4% for HPF. To exclude the possibility that EIs resulted from lack of histones in these regions, we plotted nucleosome density around EIs, and no depletion of nucleosomes was observed in EIs (Additional file 7: Figure S5), indicating that the observation of EIs is not due to nucleosome positioning.

**Table 1:** Overlap of EIs with CpG islands (CGIs) and transcription start sites (TSSs)

**Cell line**	**Number of EIs**	**Average Size of EIs (bp)**	**% of EIs overlap CGIs (En.**^**b**^**, P**^**c**^**)**	**% of EIs overlap TSSs (En.**^**b**^**, P**^**c**^**)**	**# of genes associated with EIs**^**a**^
H1	1,060	4880	9.7 (1.3, 0.06)	7.9 (5.0, <10^-3^)	119
ADA-38	758	4401	4.7 (0.6, 0.99)	4.6 (2.7, <10^-3^)	60
HA	2,254	5029	14.2 (1.8, <10^-3^)	12.4 (7.8, <10^-3^)	338
HAEC	2,359	5867	13.6 (1.6, <10^-3^)	11.3 (5.4, <10^-3^)	373
HPF	2,465	5477	17 (2.1, <10^-3^)	12.7 (6.5, <10^-3^)	409

^**a**^ 1 kb up and downstream of TSS overlapping EIs; ^b^ Enrichment compared to random patterns; ^c^ p values calculated by 1,000 permutations.

Among the five cell lines, 4.6% to 12.7% of EIs coincided with transcriptional start sites (TSSs), which associated with 60 to 409 genes across cell types. Compared to random, the enrichment at TSS ranged from 2.7 (in ADA-38) to 7.8 (HA), with randomization p-values < 10^-3^ for all cell lines (Table 1). We further investigated the spatial relationship between EI and CpG islands (CGI). We found that 4.7% to 17% of EIs overlapped with CGIs, with enrichment ranging from 0.6 to 2.1. The randomization test suggested that EIs significantly overlapped with CGI in differentiated cells, but not in ES and iPS cells (Table 1).

To probe the chromatin features of EIs, we compared locations of EIs in H1, HAEC and HPF with public datasets of comparable cell lines [10,23]. Interestingly, EIs highly coincide with regions interacting with CCCTC-binding factor (CTCF), the major organizer of higher-order chromatin in mammalian genomes (Figure 2). Overall, up to 61.3% of EIs overlap with CTCF binding regions, which are 8.2-fold enriched compared with the random pattern (P < 10^-3^, Table 2). Furthermore, up to 49% of EIs overlap with DNase hypersensitive sites (DHSs), the hallmark of open chromatin, which is 6.5-fold enrichment compared with the random (P < 10^-3^, Figure 2 and Table 2). We further explored the overlaps of EIs with other histone modifications, and found that EIs highly overlaps with H3K4me3 (Enrichment up to 5.3) and H3K9ac (Enrichment up to 3.3), but less enrich for H3K27me3 (Enrichments from 1.7 to 2.2) and H3K36me3 (Enrichments from 0.7 to 2.1). The enrichments are similar among the three cell types. In addition, we investigated the enrichment by comparing EIs with random pattern within LOCK regions, and got similar results and even stronger enrichments for CTCF (up to 13.9 fold, Table 2).

**Table 2:** **Overlaps (%) of EIs with chromatin marks**^**a**^

	**EIs overlap with**	**Observed**	**Random within LOCKs**	**Fold enriched**^**c**^	**P value**^**b**^	**Random at WG**	**Fold enriched**^**c**^	**P value**^**b**^
HAEC	CTCF	61.3	4.4	13.9	<10^-3^	7.5	8.2	<10^-3^
	DHSs	48.9	10.1	4.8	<10^-3^	7.5	6.5	<10^-3^
	H3K4me3	23.3	6.8	3.4	<10^-3^	5.6	4.2	<10^-3^
	H3K27me3	46.0	34.1	1.4	<10^-3^	27.8	1.7	<10^-3^
	H3K36me3	20.0	14.1	1.4	<10^-3^	9.7	2.1	<10^-3^
	H3K9ac	17.7	6.0	2.9	<10^-3^	5.3	3.3	<10^-3^
H1	CTCF	46.9	9.0	5.2	<10-3	11.8	4.0	<10^-3^
	DHSs	36.0	7.1	5.1	<10-3	18.1	2.0	<10^-3^
	H3K4me3	13.2	1.8	7.4	<10-3	1.4	9.8	<10^-3^
	H3K27me3	21.6	10.8	2.0	<10-3	9.8	2.2	<10^-3^
	H3K36me3	11.0	16.1	0.7	1	15.2	0.7	1
	H3K9ac	28.9	11.8	2.4	<10-3	11.5	2.5	<10^-3^
HPF	CTCF	56.9	7.9	7.2	<10^-3^	9.6	5.9	<10^-3^
	DHSs	71.0	27.7	2.6	<10^-3^	19.2	3.7	<10^-3^
	H3K4me3	30.8	8.1	3.8	<10^-3^	13.0	2.4	<10^-3^
	H3K27me3	53.1	34.2	1.6	<10^-3^	29.0	1.8	<10^-3^
	H3K36me3	22.6	16.5	1.4	<10^-3^	24.9	0.9	0.85
	H3K9ac	13.2	2.5	5.2	<10^-3^	6.2	2.1	<10^-3^

^a^ EIs of HAEC, H1 and HPF were compared with chromatin marks of HUVEC, H1 and normal lung fibroblasts, respectively (ref.10).

^b^P values were calculated by 1000 permutations.

^c^Enrichment is calculated as the ratio of observed to random.

**Figure 3:**
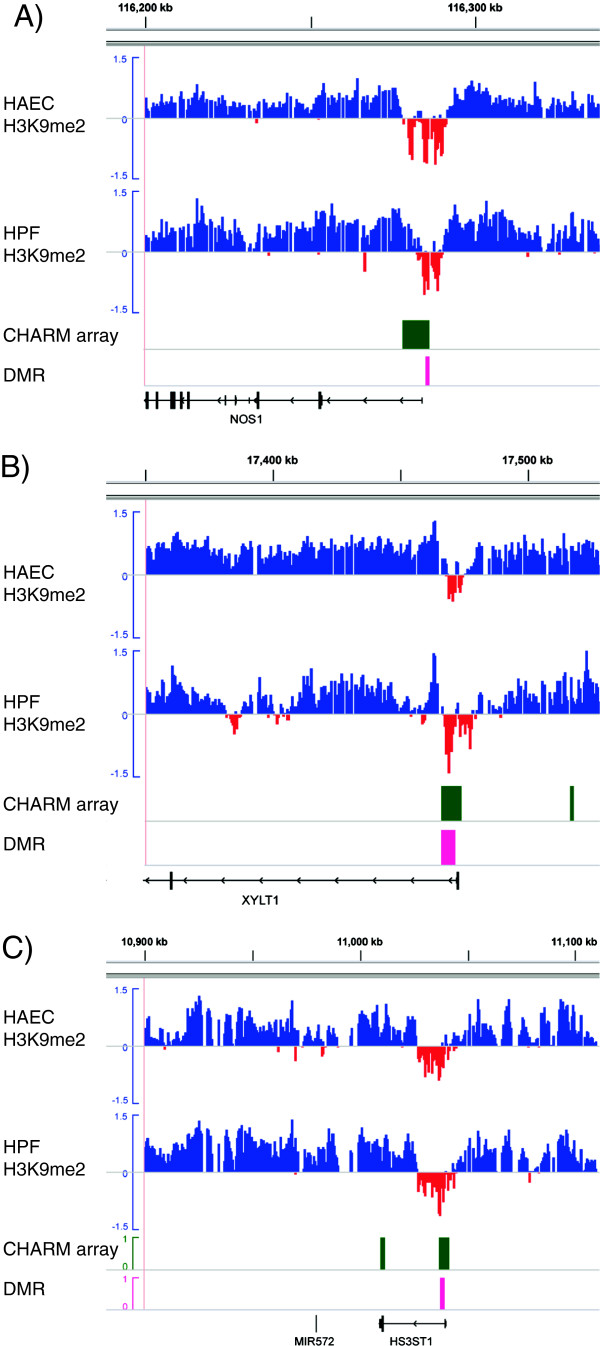
**EIs are enriched for differential methylation regions (DMRs).** The H3K9me2 signals of HAEC and HPF are compared with regions of T-DMRs (pink bars). Regions of CHARM array are denoted by green bars. EIs (red dips) clearly overlap T-DMRs near the TSSs of *NOS1* (**A**), *XYLT1* (**B**) and *HS3HT1* (**C**).

We then asked whether there is any association between EIs and DMRs. For this purpose, we compared genomic locations of EIs with DMRs identified by CHARM array [17,18]. We found that EIs are highly enriched for DMRs distinguishing tissue types (T-DMRs). For example, EIs near TSSs of nitric oxide synthase 1 (*NOS1*), xylosyltransferase I (*XYLT1*) and heparan sulfate (glucosamine) 3-O-sulfotransferase 1 (*HS3ST1*) all overlap T-DMRs (Figure 3). Overall, a large fraction of EIs (39-62% across the five cell types) overlap with T-DMRs, with enrichment from 2.1 to 2.9 folds relative to random patterns (Table 3; p < 10^-3^).

**Table 3:** Percentage of EIs overlap with DMRs

**Cell line**	**Overlaps with DMRs (%)**																				
	**T-DMR**			**R-DMR**						**C-DMR**						**Whole genome C-DMR**					
				**Hyper**			**Hypo**			**Hyper**			**Hypo**			**Hyper**			**Hypo**		
	**%**	**En.**	**p**	**%**	**En.**	**p**	**%**	**En.**	**p**	**%**	**En.**	**p**	**%**	**En.**	**p**	**%**	**En.**	**p**	**%**	**En.**	**p**
H1	53.4	2.8	<10^-3^	19.4	3.1	<10^-3^	5.8	3.1	0.02	11.7	3.8	<10^-3^	4.9	1.4	0.24	5.6	5.2	<10^-3^	0.9	1	0.31
ADA-38	39.1	2.1	<10^-3^	17.4	3.2	<10^-3^	2.2	1.2	0.33	6.5	2.2	0.05	6.5	2	0.08	4.1	4.1	<10^-3^	0.4	0.5	0.84
HA	60.4	2.9	<10^-3^	19.3	3.1	<10^-3^	11	5.5	<10^-3^	16.7	5.4	<10^-3^	4.5	1.3	0.25	8.6	7.3	<10^-3^	1	1.1	0.34
HPF	59.5	2.8	<10^-3^	17.5	2.8	<10^-3^	12.8	6.1	<10^-3^	17.5	5.1	<10^-3^	5.7	1.5	0.09	9.4	7.6	<10^-3^	1.3	1.4	0.04
HAEC	62.3	2.9	<10^-3^	14.3	2.2	<10^-3^	11.7	5.6	<10^-3^	15.7	4.5	<10^-3^	4.6	1.2	0.29	7	5.3	<10^-3^	1.1	1.1	0.27

Notes: En. = Enrichment. Statistical significances were tested by 1000 permutations.

We further tested the relationship between EIs and DMRs associated with reprogramming (R-DMRs). Similar to T-DMRs, R-DMRs were more methylated in iPSC cells compared to fibroblasts (Hyper R-DMRs) were also significantly enriched in EIs of all the cell types, whose enrichments ranging from 2.2 to 3.2 fold. However, R-DMRs less methylated in iPSC cells (Hypo R-DMRs) were highly enriched in EIs of differentiated cells (5.1 to 6.2 folds enrichment, P values all < 10^-3^), but much less enriched in PSCs (1.2 to 3.1 folds of enrichment). Importantly, EIs in HPF, the same cell type of parental cells in reprogramming, are strongly enriched for hypomethylated R-DMRs (enrichment = 6.1, P < 10^-3^), whereas those in iPSCs did not significantly overlap with hypomethylated R-DMRs (enrichment = 1.2, P = 0.33), indicating a coordinated hypomethylation in these EIs during reprogramming.

We then compared EI locations with colon cancer-associated DMRs (C-DMRs) and observed an opposite trend to that of R-DMRs. EIs in 4 out of 5 cell lines were significantly enriched for C-DMRs more methylated in colon cancers (hypermethylated C-DMRs), and the enrichment ranged from 3.8 to 5.4 fold (Table 3). In contrast, all five cells types were not significantly enriched for C-DMRs less methylated in cancers (hypomethylated C-DMRs). These results were further confirmed by comparing EIs with an independent list of C-DMRs discovered by whole genome bisulfite sequencing [20]. EIs of all five cell lines significantly overlapped hypermethylated C-DMRs (enrichment from 4.1 to 7.6 fold, P values all < 10^-3^), whereas none of them were significantly enriched for hypomethylated C-DMRs (Table 3). Interestingly, almost all EIs (98%) that associated with hypermethylated C-DMRs also overlap CGIs. These data suggest that EIs in normal cells may become hypermethylated in cancers.

**Figure 4:**
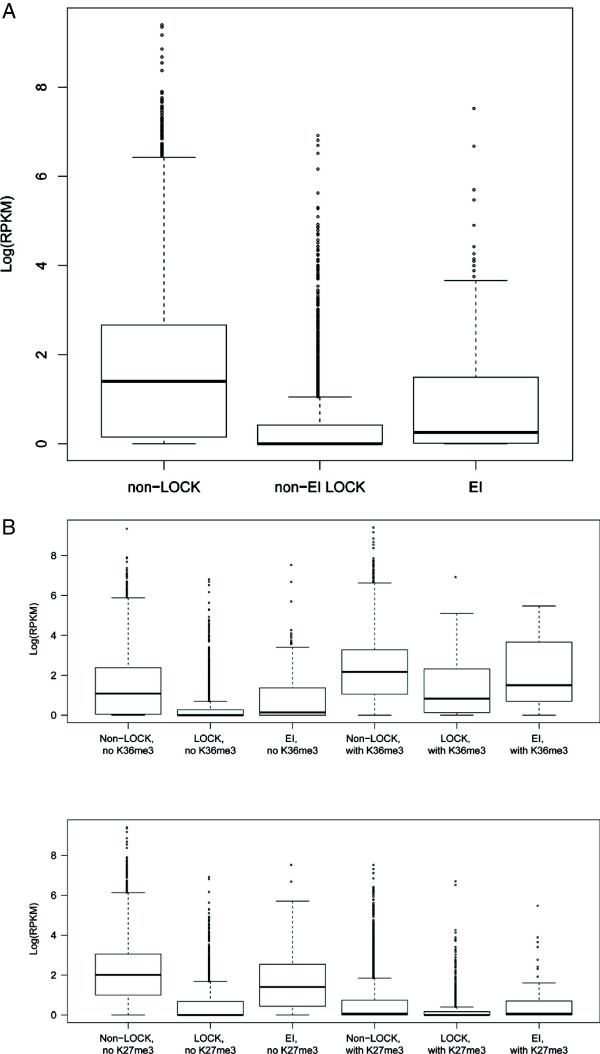
**Expression of genes associated with EIs.** We compared expression levels for genes with TSS at different regions. Expression values are RPKM (read per kb per million reads) for lung fibroblast IMR90 [7]. **A**) boxplot of expression level of genes with TSS 1) overlapping EIs; 2) overlapping LOCKs but not EIs; and 3) not overlapping EIs. **B**) Relationship between H3K36me3/H3K27me3 and expression of EI associated genes.

To explore the biological role of EIs, we compared expression levels of genes associated with EIs, of genes with LOCKs but not EIs, and of genes not overlapping LOCKs (Figure 4A). It is clear that expressions of genes overlapping EIs are significantly higher than those of within LOCKs but not EIs (*t*-test, p < 2–16). To further test whether EI associated genes are regulated by other histone marks, we investigated the relationship between H3K36me3/H3K27me3 and genes with EIs, with LOCKs and without LOCKs (Figure 4B). In either category (with or without K36me3/K27me3), genes at LOCK regions always have the lowest expression and genes at non-LOCK regions have the highest. However, genes with EIs have expressions in the middle, and positively (negatively) associated with H3K36me3 (H3K27me3), indicating that EI related genes could be regulated by these two marks.

Then we conducted Gene Ontology (GO) analysis with genes whose TSSs are associated with EIs. EI-associated genes in differentiated cells were strongly associated with 1) biological processes such as system development, cell adhesion and cell differentiation; 2) cellular compartments of plasma membrane and synapse, and 3) molecular function of ion binding and channel activity (Table 4).

**Table 4:** Top 10 GO terms of genes associated with EIs

**GO terms**	**% of genes**	**Fold en.**	**FDR**
**Biological processes**			
nervous system development	12.6	2.2	9E-11
developmental process	25.5	1.6	4E-09
system development	20.5	1.7	4E-09
anatomical structure development	21.5	1.6	1E-08
multicellular organismal development	23.4	1.6	2E-08
cell adhesion	8.7	2.4	8E-08
biological adhesion	8.7	2.4	8E-08
cell differentiation	15.2	1.8	4E-07
cell development	8.0	2.4	4E-07
multicellular organismal process	31.1	1.4	4E-07
**Cellular compartment**			
plasma membrane part	21.1	1.9	4E-14
plasma membrane	30.3	1.6	1E-12
membrane part	44.2	1.3	1E-09
integral to plasma membrane	12.6	2.1	3E-09
intrinsic to plasma membrane	12.7	2.1	5E-09
membrane	47.0	1.3	2E-08
intrinsic to membrane	37.3	1.4	2E-07
integral to membrane	35.9	1.4	1E-06
synapse	5.3	3.0	2E-06
extracellular region	16.7	1.7	6E-06
**Molecular function**			
calcium ion binding	9.7	2.1	4E-06
gated channel activity	4.5	2.9	9E-05
molecular transducer activity	17.5	1.5	6E-04
signal transducer activity	17.5	1.5	6E-04
substrate specific channel activity	5.1	2.5	6E-04
ion channel activity	4.9	2.5	8E-04
channel activity	5.1	2.4	1E-03
passive transmembrane transporter activity	5.1	2.4	1E-03
cation channel activity	3.9	2.8	2E-03
ligand-gated channel activity	2.3	3.6	1E-02

**Figure 5:**
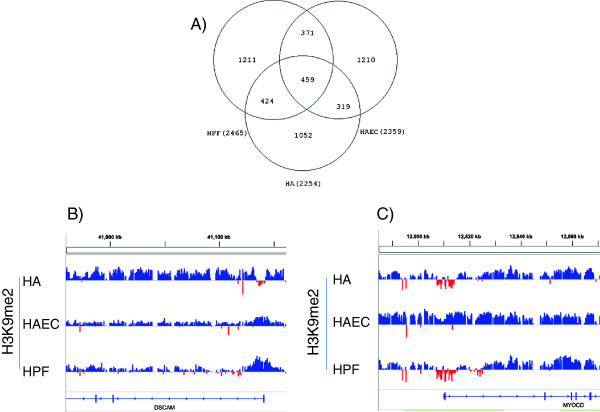
**Cell type specific EIs.A**) The Venn diagram shows overlaps of EIs among three differentiated cell lines (HA, HAEC and HPF). **B**-**C**) Shown are examples of EIs which differ among cell types. The annotation of tracks is the same as in Figures 2 and 3. An EI is found near the 5’ end of the myocardin gene (*MYOCD*) in HA and HPF but not in HAEC. An EI is found in the TSS of the Down syndrome cell adhesion molecule gene (*DSCAM*) specifically in HA but not in other two cell types (**C**).

Finally, to test whether EIs are associated with specific cellular functions, we compared the location of EIs among the three differentiated cell lines. Based on our current strategy to define EIs, ~50% of them are cell type specific (Figure 5A). It should be noted that the detection of EI is based on the definition of LOCKs as well as the amount of reduction of H3K9me2 levels within LOCK bodies. Some tissue specific EIs may be due to differential LOCKs or different amount of H3K9me2 reductions among cell types. Due to these reasons the number of tissue specific EIs is likely an over-estimate. New technology with higher resolution and dynamic range, such as ChIP-seq, will help achieve better accuracy and specificity in tissue comparisons. Nevertheless, we found that some tissue specific EIs are biologically meaningful. For example, an EI is located near the TSS of Down syndrome cell adhesion molecule gene (*DSCAM*) in astrocytes (HA) but not the other two cell types (Figure 5B). It was shown that Dscam diversity is essential for neuronal circuit assembly [24], and genetic variations of this gene were associated with Down syndrome and congenital heart disease (DSCHD) [25] and bipolar disorder [26]. Furthermore, an EI is found on the 5’ end of myocardin (*MYOCD*) gene in HA and HPF but HAEC (Figure 5C). Myocardin is a coactivator of serum response factor which specifically expressed in cardiac and smooth muscle cells [27], and promoter variation of this gene was proposed as a biomarker of cardiac hypertrophy [28]. These data suggest that EIs may be important in regulating specific cellular functions.

## Discussion

In summary, by examining the genome-wide distribution of H3K9me2 in human PSCs and differentiated cells, we found a novel microstructure within heterochromatin domains of thousands of small euchromatin islands (EIs) located within large H3K9me2 blocks (LOCKs). EIs are strongly associated with open chromatin regions (DHSs), active chromatin marks (H3K4me3 and H3K9ac) and higher-order chromatin organizers (CTCF). Furthermore, EIs are highly enriched for DMRs associated with tissue specificity (T-DMRs), reprogramming (R-DMRs) and cancers (C-DMRs). This association is particularly strong for hypomethylated R-DMRs and hypermethylated C-DMRs. Genes associated with EIs are enriched for annotations of system development, cellular differentiation and cell adhesion. These results suggested that EIs may coordinate higher order chromatin and mediate co-regulation of DNA methylation in reprogramming and tumorigenesis. However, further experimental work is needed to address the functional relevance of EIs and their strong association with CTCF and DMRs.

In this study, we compared H3K9me2 profiles with publicly available epigenomic data generated from similar cell types. This strategy may lead to biased estimation of the enrichments of EIs with other epigenetic marks, because patterns of EIs may be different between the two samples. Comparison of exactly matched cell lines and cultures could assess the association between them more accurately.

Note that a previous literature used the term “euchromatic islands” in a completely different context, simply to describe chromatin regions with H3K4me3 and CpG islands, essentially describing promoter regions of active genes [4,29]. As that term was rarely used previously, and to convey a completely different meaning, we do not think there will be confusion with our newly defined (and differently spelled) “euchromatin islands” or EIs, namely H3K9me2 depleted regions/islands within an ocean of heterochromatin (LOCKs), enriched for regulatory elements such as enhancers (DHSs) and insulators (CTCF). Thus, EIs are novel units of the genomic “tool box” which may be important in epigenetic regulation as suggested by their strong association with DMRs (Table 3).

Higher-order organization of the genome remains a highly active area to be explored. Recent evidence indicates the presence of spatial compartments of active and repressive chromatin domains as general principles of genome organization in mammalian cells [30,31], and CTCF mediates intra- and inter-chromosomal interactions by tethering chromatin regions binding CTCF [32]. It would be interesting to explore the possibility that euchromatin islands act as “anchors” for the interactions among heterochromatin domains or between heterochromatic and enchromatic regions. Moreover, the relationships between EIs and heterochromatin formation, and the biophysical features of EIs are interesting questions for future investigation.

Although evidence provided in this and other studies have indicated that large heterochromatin domains are highly dynamic in stem cell differentiation and tumorigenesis [11-15], Lienert et al. indicated that genome coverage of H3K9me2 domains do not increase globally during neuronal differentiation of mouse ES cells [33]. First of all, lineage specificity of differentiated cells may explain the conflicts. As reported in our earlier work [11] the amount of LOCKs detected from brain and ES cells are comparable (9.8% vs. 4%), whereas the amount is very high in liver (45.6%). The Lienert study used in vitro differentiated neurons as differentiated cells which is more similar to brain*.* Furthermore, the inconsistence may be due to sensitivities of different statistical methods for finding large domains, heterogenenity of stem cells, and so on. Notably, extensive deduction of LOCKs during EMT suggested that quantitative differences of these large domains may be functionally important [13]. Nevertheless, further studies on homogenous stem cell populations may be helpful to address these debates. Whatever it holds, functionally investigations of these large domains should provide important insight toward how higher-order chromatin affects normal development and disease.

## Conclusions

In conclusion, we have explored the microstructure of LOCKS and indentified thousands of euchromatin islands (EIs), which may be served as a finer layer of epigenomic architecture within large heterochromatin domains. The strong association of EIs with CTCF sites, DNAse hypersensitivies sites, and DMRs suggests that EIs play an important role in normal epigenomic architecture and its disruption in disease.

## Methods

### Cell culture

Human H1 ESCs and ADA-38 iPSCs were cultured as described [34]. Primary Human Pulmonary Fibroblasts (HPF), Human Aortic Endothelial Cells (HAEC) and Human Astrocytes (HA) were purchased from ScienCell Research Laboratories (San Diego, CA), and cultured as recommended by ScienCell.

### ChIP-chip

ChIP-chip experiments were performed as described [11], using a commercial monoclonal antibody (Abcam, ab1220), which specifically recognizes H3K9me2 but not other modifications [35]. The passage numbers for cells used for ChIP analysis were P46 for H1, P59 for ADA-38 and P2 for primary cells from ScienCell. We first mapped whole genome distribution of H3K9me2 using “Mouse ChIP-chip 2.1M Economy Whole-Genome Tiling arrays (4 arrays per set) from NimbleGen”, with 203 bp of median probe spacing. Then we repeated the microarray experiments on one of the Mouse ChIP-chip 2.1 M Whole-Genome Tiling sets, whose median probe spacing is 100 bp. The replicate array covers 10% of the genome, including part of chromosome 6 (111,920,005-170,893,515), whole chromosome 7 and part of chromosome 8 (521–74,730,105). For the replicate experiments, cell cultures, ChIP sample preparation, labeling and hybridization were performed independently.

### ChiP-chip data analysis

Data were first normalized by partial quantile normalization, then LOCKs were detected based on the smoothing values of normalized log2 ratios of data between ChIP and input channels [11]. The euchromatin islands (EIs) are defined as short regions within LOCK body that have low H3K9me2 methylation levels. To detect such regions we designed the following smoothing based approach. The log2 ratios for probes within LOCKs were first smoothed using 5000 bp window. The relatively short smoothing window is used to capture the signal variations in small regions. Genomic regions with smoothed value less than 1% of all the smoothed values were defined as EIs. It is required that EIs are at least 1000 bps long and contain at least 10 probes. EIs less than 1000 bps apart will be merged into one. It is also required that the EIs are at least 20000 bps away from the LOCK boundaries. This is because the log2 ratios are smaller at LOCK boundaries. Such requirement prevents mistakenly taking LOCK boundaries as EIs. A flow diagram showing the algorithm for detecting EIs was provided in Additional file 8: Figure S6. Microarray data have been submitted to GEO database (accession numbers: GSE37335).

To compute the enrichment of EI overlapping other genomic features (CTCF, DHS, etc.), we first calculated the percent of EIs overlapping the feature. Then a set of genomic regions was randomly sampled. The number and lengths of the random regions match the EI list. The random regions were then compared with the feature to obtain a percentage of overlapping. Such process was repeated 1000 times. The percentages obtained from the process form the null distribution for percentage of overlapping. The p-values and enrichments were computed based on the null distribution. The p-values were then corrected for multiple testing using Bonferroni correction. Publicly available datasets used for analysis were listed in Additional file 9: Table S3.

### Quantitative PCR (qPCR)

Experiments of qPCR were conducted as described [11]. Primer sequences are provided in Additional file 10: Table S4.

### GO analysis

GO analysis was performed using DAVID tools as described [36], using the list of genes overlapping EIs of the three differentiated cell lines (HA, HAEC and HPF).

## Abbreviations

EI: Euchromatin island; DHS: DNase hypersensitive site; DMR: Differential methylation region; H3K9me2: H3 lysine 9 dimethylation; LOCK: Large organized chromatin k9-modification; ESC: Embryonic stem cell; PMD: Partial methylated domains; PSC: Pluripotent stem cell; HA: Human astrocytes; HAEC: Human aortic endothelial cell; HPF: Human pulmonary fibroblast; TSS: Transcription start site; CTCF: CCCTC-binding factor.

## Competing interests

The authors declare that they have no competing interests.

## Authors' contributions

BW and APF conceived the project and designed the study. BW performed cell culture of primary cells, ChIP and qPCR; HW performed data analysis; YL and GQD generated PSC lines; BE conducted microarray analysis; BW, HW and APF prepared the manuscript. All authors read and approved the final manuscript.

## Supplementary Material

Additional file 1 **Table S1.** Description: Genome coverage and average size of LOCKs in human PSCs and differentiated cells. Click here for file

Additional file 2 **Figure S1(A-D).** Description: qRCR validation of H3K9me2 ChIP-chip data on 23 loci. Upper panels show log2 (ChIP/Input) ratios of microarrays and green bars denote regions selected for qPCR validation; lower panels present qPCR enrichments of ChIP over input in the selected regions. Click here for file

Additional file 3 **Figure S2.** Description: LOCKs overlap partial methylation domains (PMDs). (A) One representative region (on chromosome 17) where LOCKs and PMDs overlap, green and orange bars show locations of LOCK (green) and PMD (orange), and hypomethylation blocks (purple), respectively; (B) H3K9me2 density in and out of PMDs. X-axis is the probe log2 ratios between ChIP and control samples. Y-axis is the the probability density. Click here for file

Additional file 4 **Table S2.** Description: Coordinates of EIs (HG18). Click here for file

Additional file 5 **Figure S3.** Description: Average H3K9me2 densities in EIs and their adjacent regions. Click here for file

Additional file 6 **Figure S4.** Description: H3K9me2 ChIP-chip experiments in whole genome (WG) and replicate (rep) arrays. Click here for file

Additional file 7 **Figure S5.** Description: Nucleosome density in EIs and adjacent regions. We compared common EIs of HA, HAEC and HPF with nucleosome maps of GM12878 (Supplementary Table S3), to overcome potential lineage specificity among those cell types. Click here for file

Additional file 8 **Figure S6.** Description: Flow diagram of EI detection. Click here for file

Additional file 9 **Table S3.** Description: Public datasets used for analysis. Click here for file

Additional file 10 **Table S4.** Description: qPCR primer sequences. Click here for file
