# Supplementary material for: Euchromatin islands in large heterochromatin domains are enriched for CTCF binding and differentially DNA-methylated regions
Source: BMC Genomics. 2012 Oct 26;13:566. doi: 10.1186/1471-2164-13-566 (PMC3507770; doi:10.1186/1471-2164-13-566)
Supplement: Additional file 1 — Table S1. Description: Genome coverage and average size of LOCKs in human PSCs and differentiated cells. [file 1471-2164-13-566-S1.docx]

Supplementary Table 1. Genome coverage and average size of LOCKs in human PSCs and differentiated cells.

| Quantiles | H1 | ADA-38 | HA | HPF | HAEC |
| --- | --- | --- | --- | --- | --- |
| **Coverage (%)** | | | | | |
| 0.5 | 87.8 | 87.1 | 87.6 | 87.1 | 88.5 |
| 0.8 | 47.9 | 39.2 | 62.8 | 62.6 | 64.2 |
| 0.9 | 24 | 17.5 | 39.3 | 44.8 | 43.4 |
| 0.95 | 9.8 | 6.7 | 15.3 | 26.9 | 21.4 |
| **Average size (kb)** | | | | | |
| 0.5 | 992.9 | 914.9 | 1135.3 | 1022.4 | 1027.0 |
| 0.8 | 272.3 | 204.7 | 414.8 | 480.6 | 470.1 |
| 0.9 | 170.5 | 141.8 | 233.3 | 315.1 | 293 |
| 0.95 | 125.5 | 102.9 | 137.1 | 219.7 | 182.4 |
