# Supplementary figures and images for: Euchromatin islands in large heterochromatin domains are enriched for CTCF binding and differentially DNA-methylated regions
Source: BMC Genomics. 2012 Oct 26;13:566. doi: 10.1186/1471-2164-13-566 (PMC3507770; doi:10.1186/1471-2164-13-566)

Fig. S1A

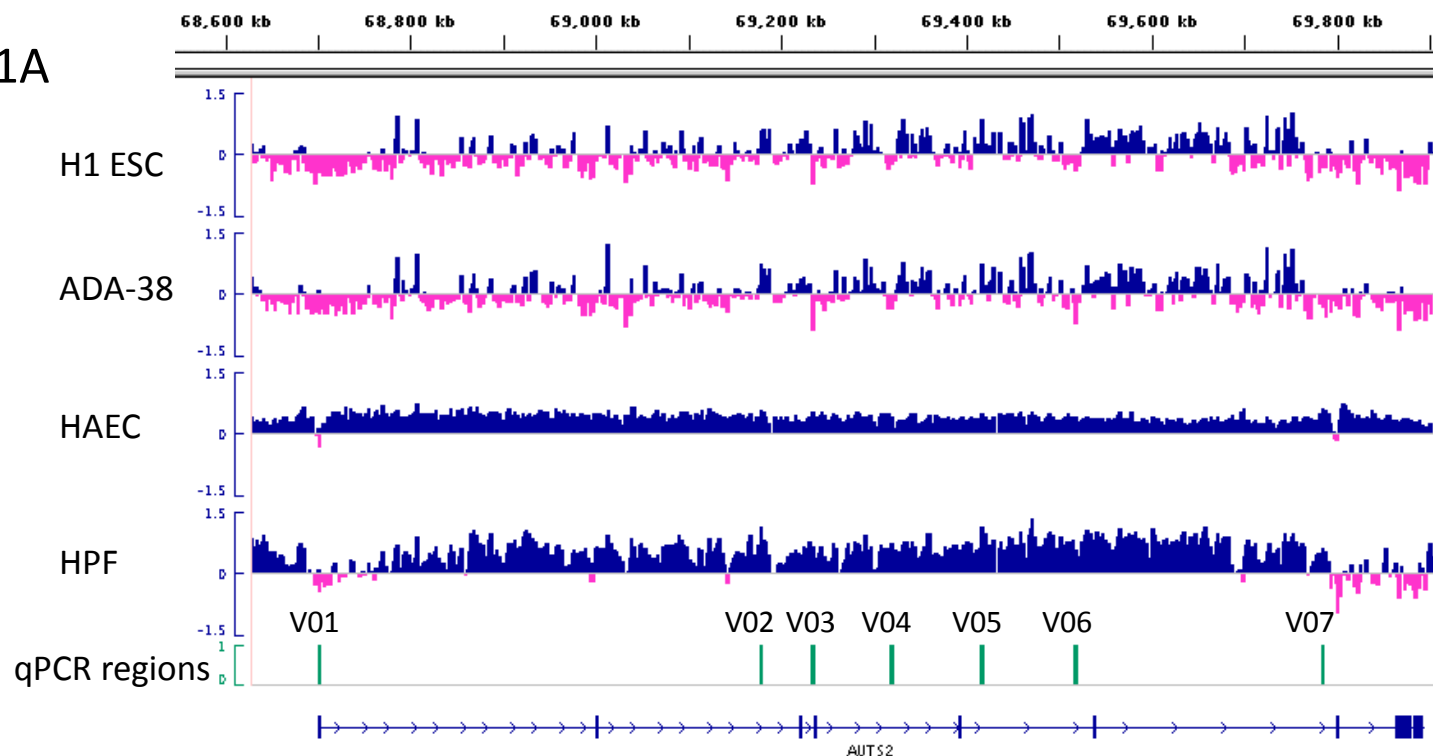

## qPCR Validation

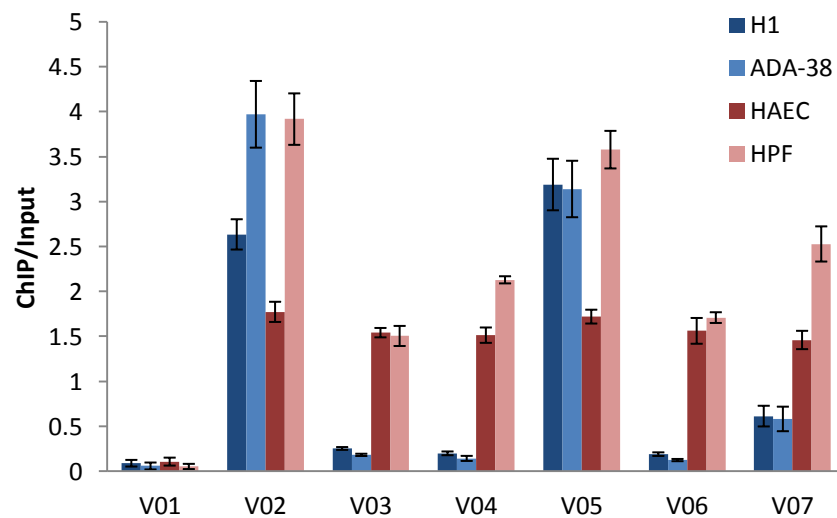

Fig. S1B

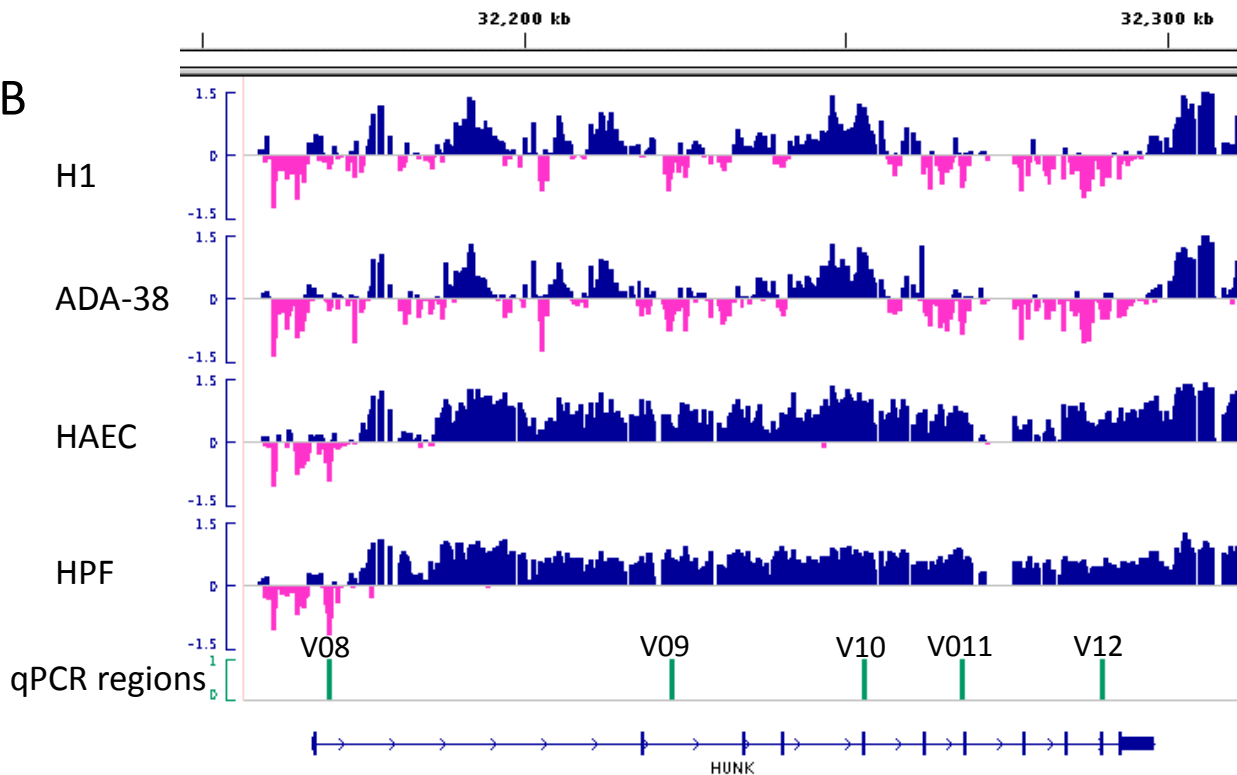

### qPCR Validation

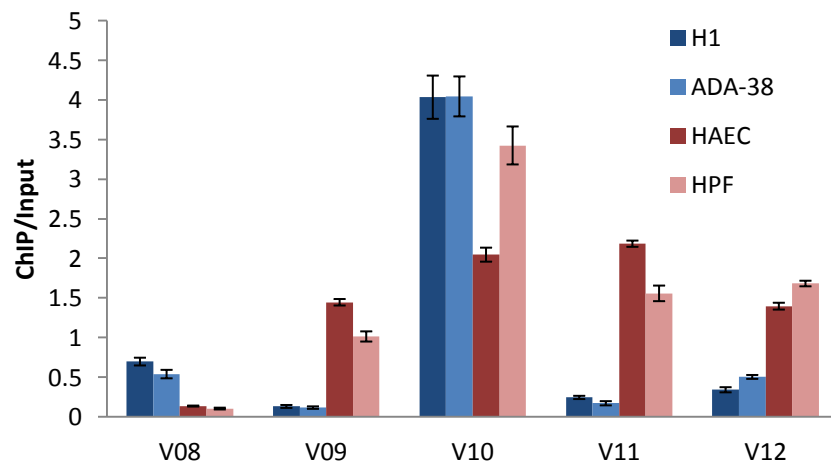

Fig. S1C

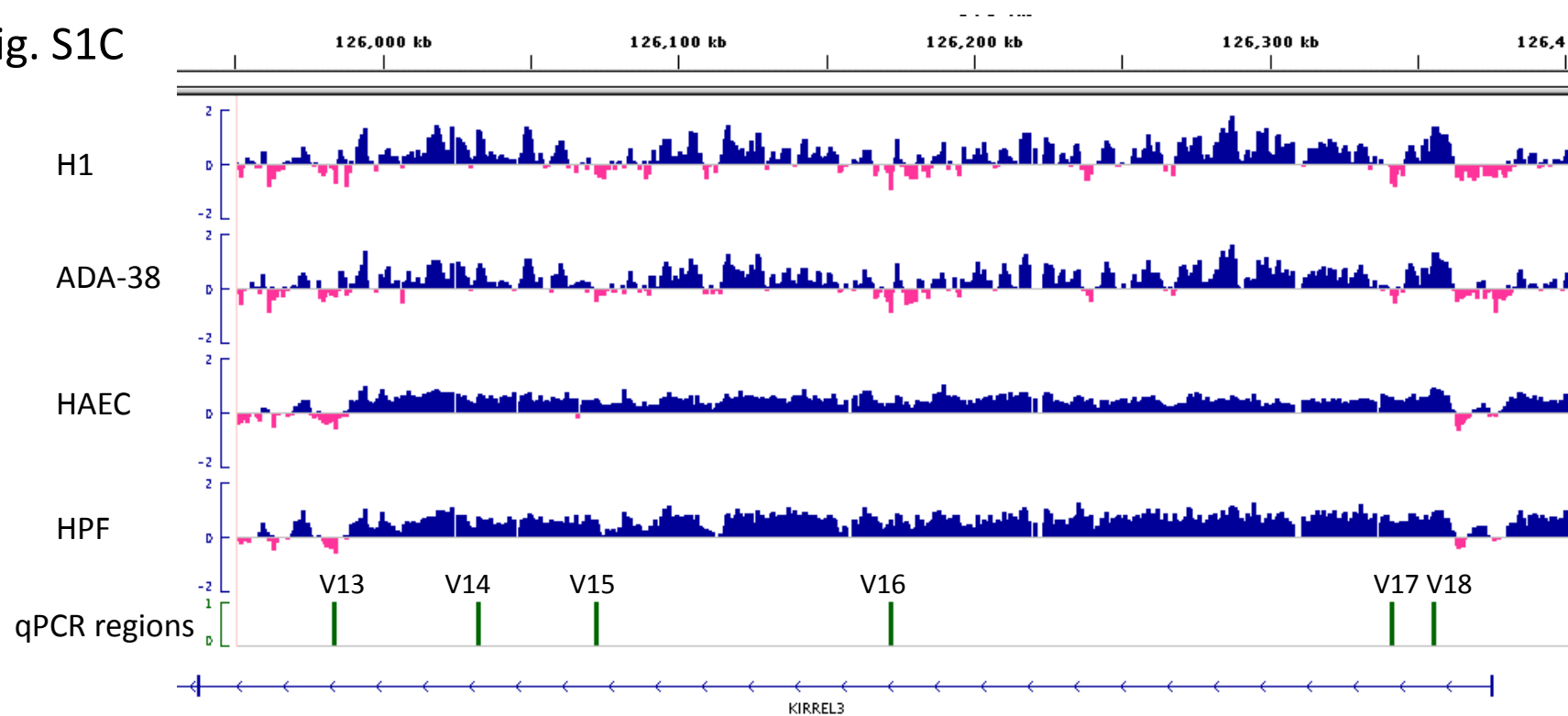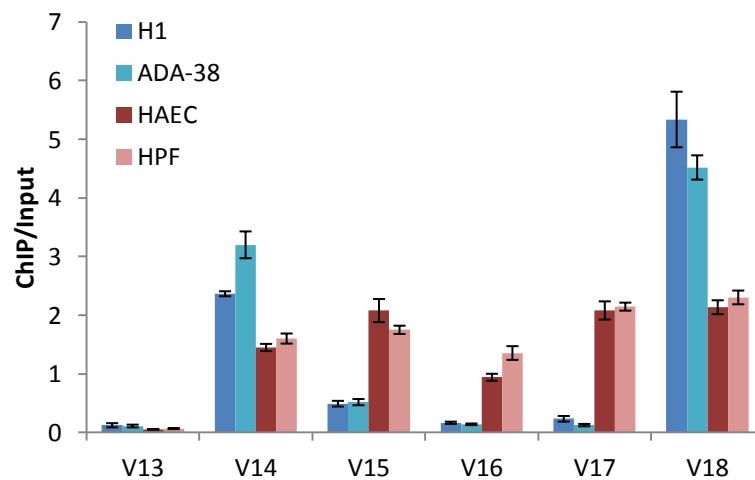

Fig. S1D

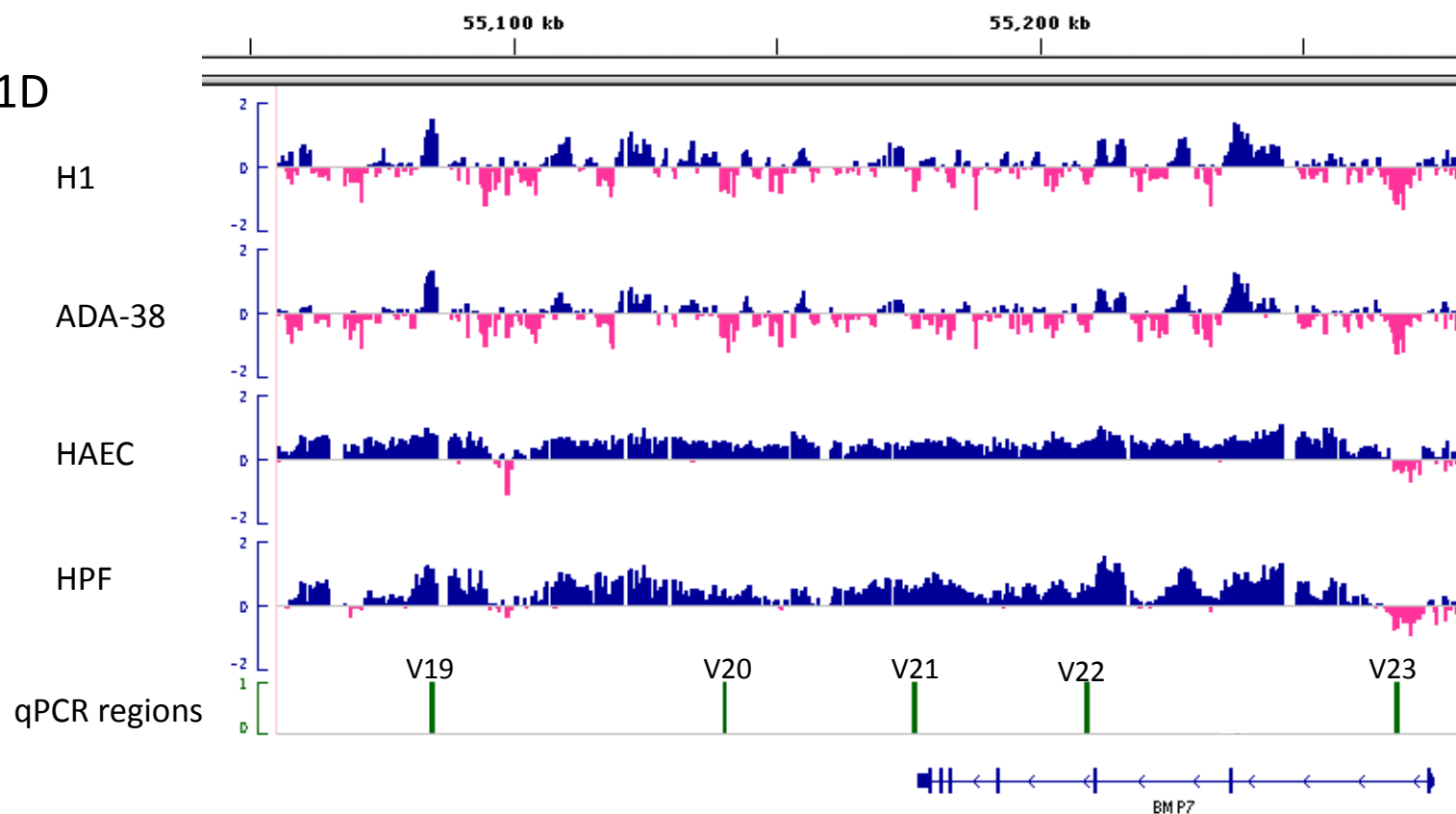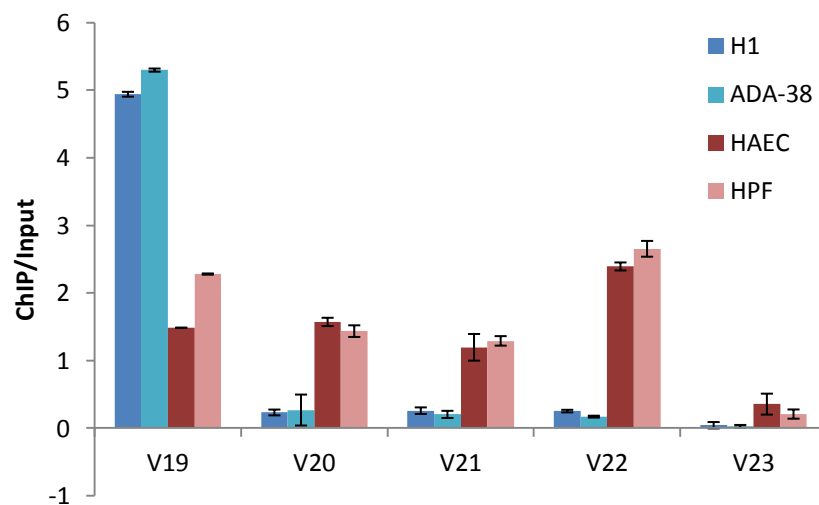

Supplement: Additional file 2 — Figure S1(A-D). Description: qRCR validation of H3K9me2 ChIP-chip data on 23 loci. Upper panels show log2 (ChIP/Input) ratios of microarrays and green bars denote regions selected for qPCR validation; lower panels present qPCR enrichments of ChIP over input in the selected regions. [file 1471-2164-13-566-S2.pdf]

Fig. S2A

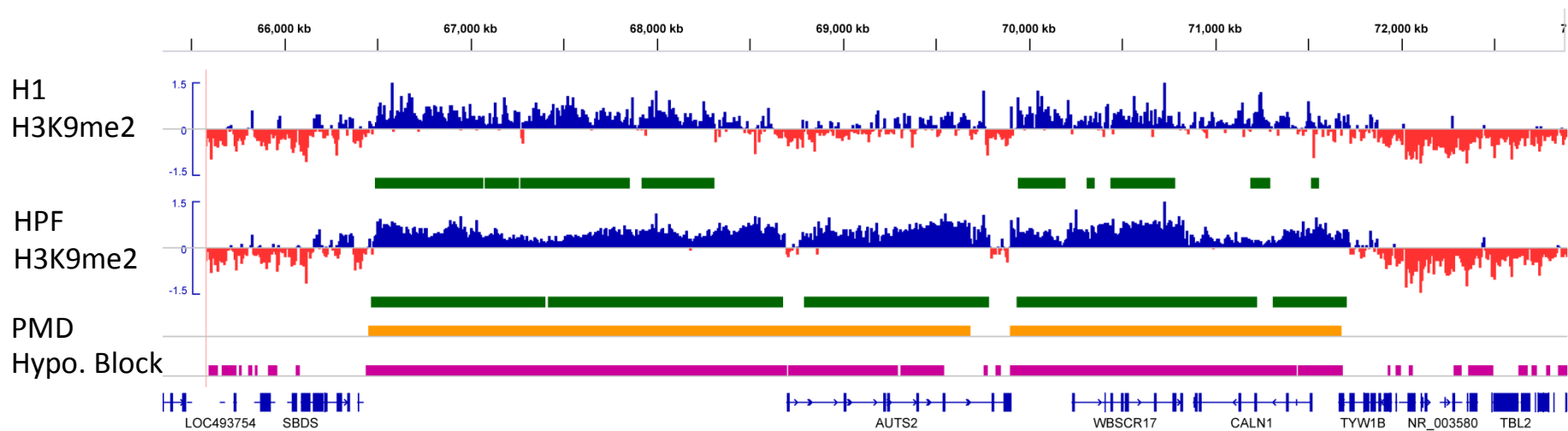

Fig. S2B

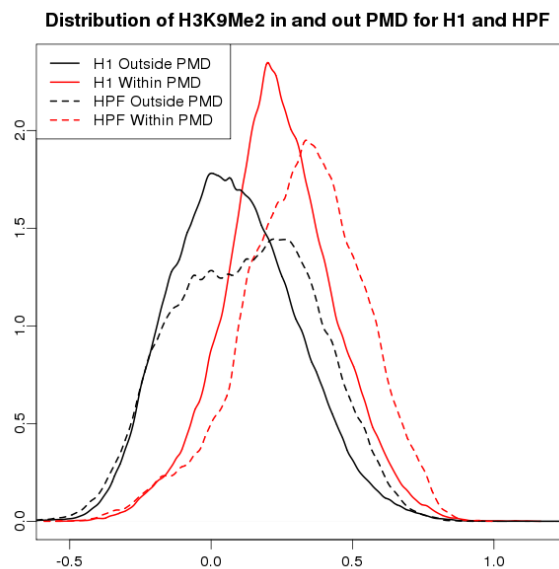

Supplement: Additional file 3 — Figure S2. Description: LOCKs overlap partial methylation domains (PMDs). (A) One representative region (on chromosome 17) where LOCKs and PMDs overlap, green and orange bars show locations of LOCK (green) and PMD (orange), and hypomethylation blocks (purple), respectively; (B) H3K9me2 density in and out of PMDs. X-axis is the probe log2 ratios between ChIP and control samples. Y-axis is the the probability density. [file 1471-2164-13-566-S3.pdf]

Fig. S3

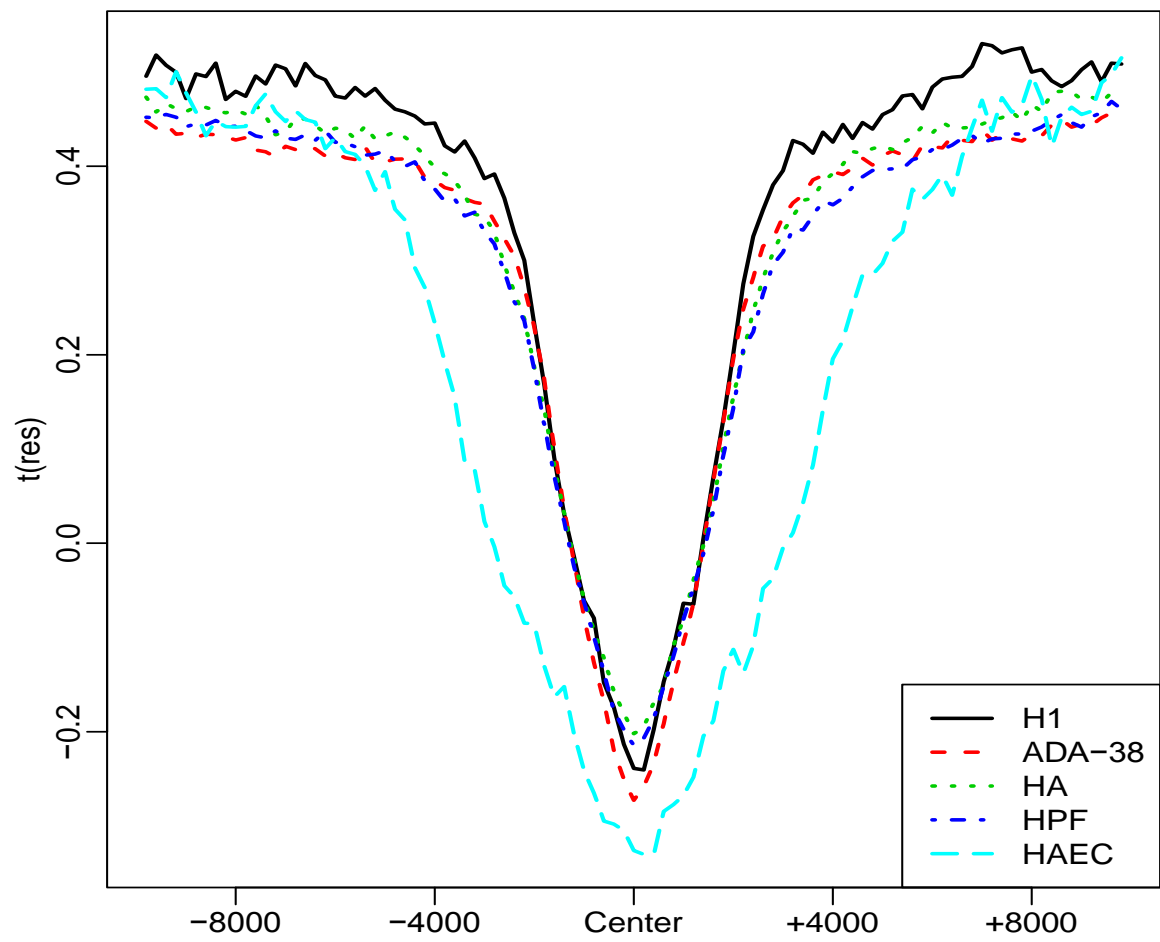

Supplement: Additional file 5 — Figure S3. Description: Average H3K9me2 densities in EIs and their adjacent regions. [file 1471-2164-13-566-S5.pdf]

Fig. S4A

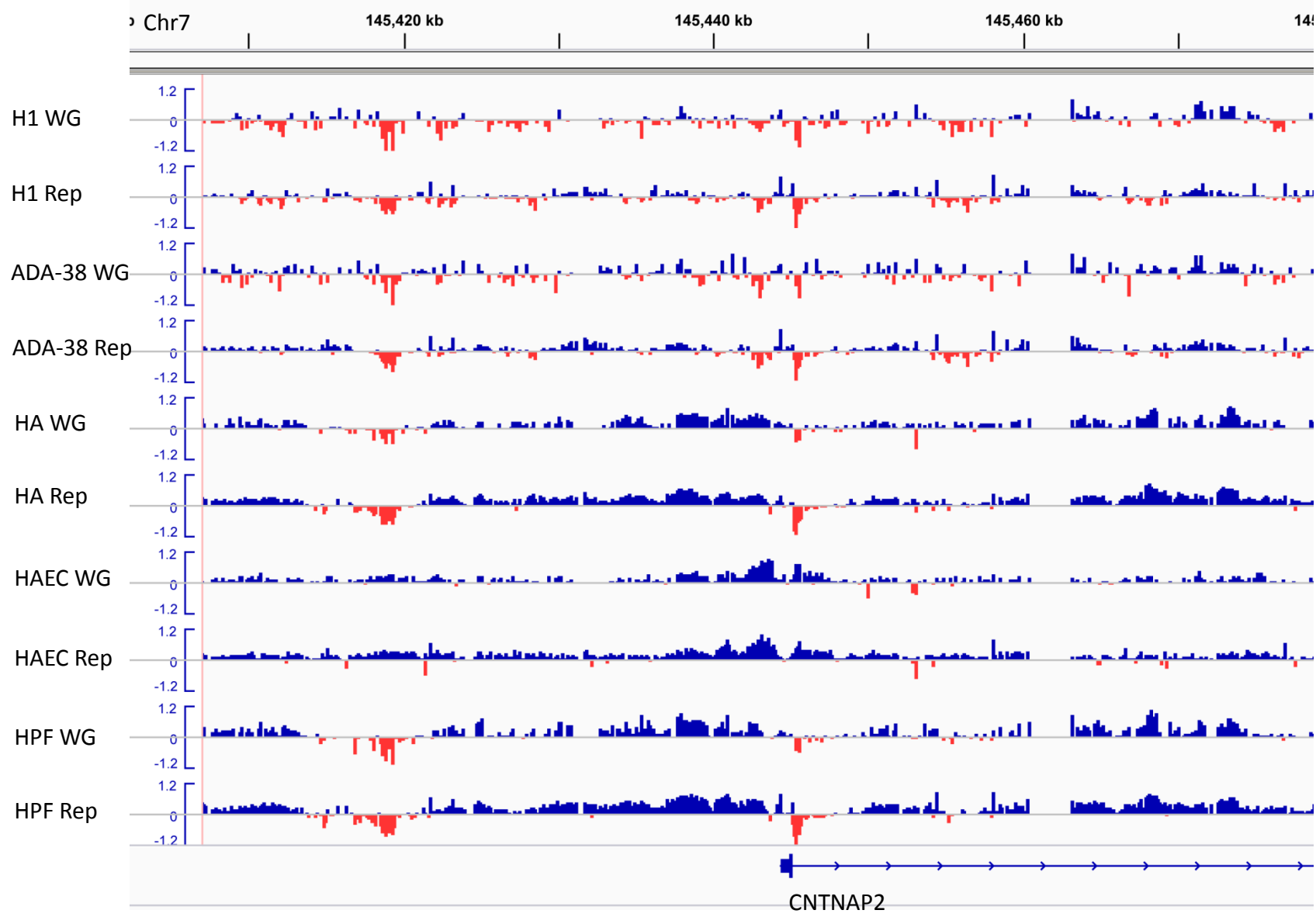

Fig. S4B

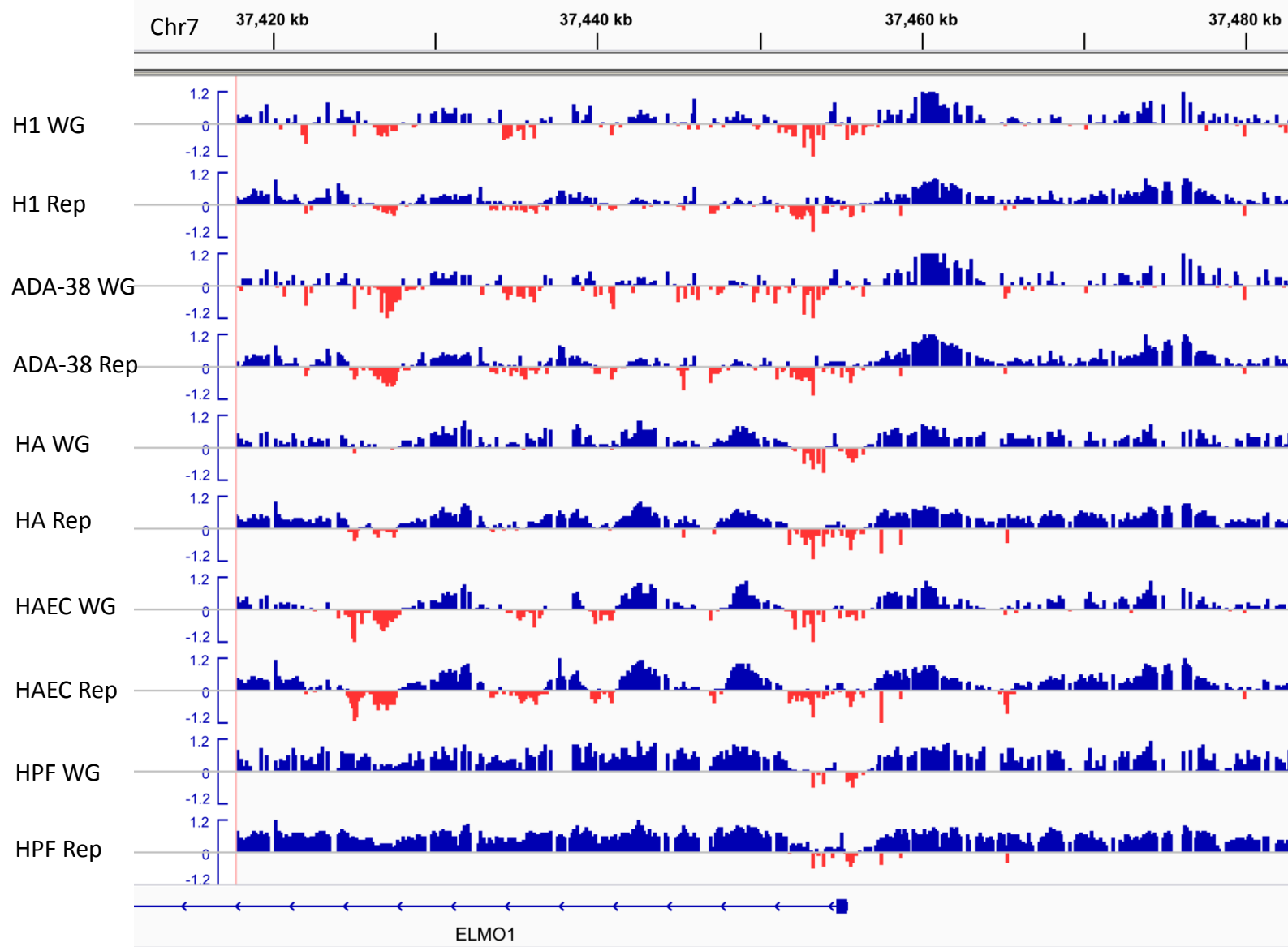

Supplement: Additional file 6 — Figure S4. Description: H3K9me2 ChIP-chip experiments in whole genome (WG) and replicate (rep) arrays. [file 1471-2164-13-566-S6.pdf]

Fig. S5

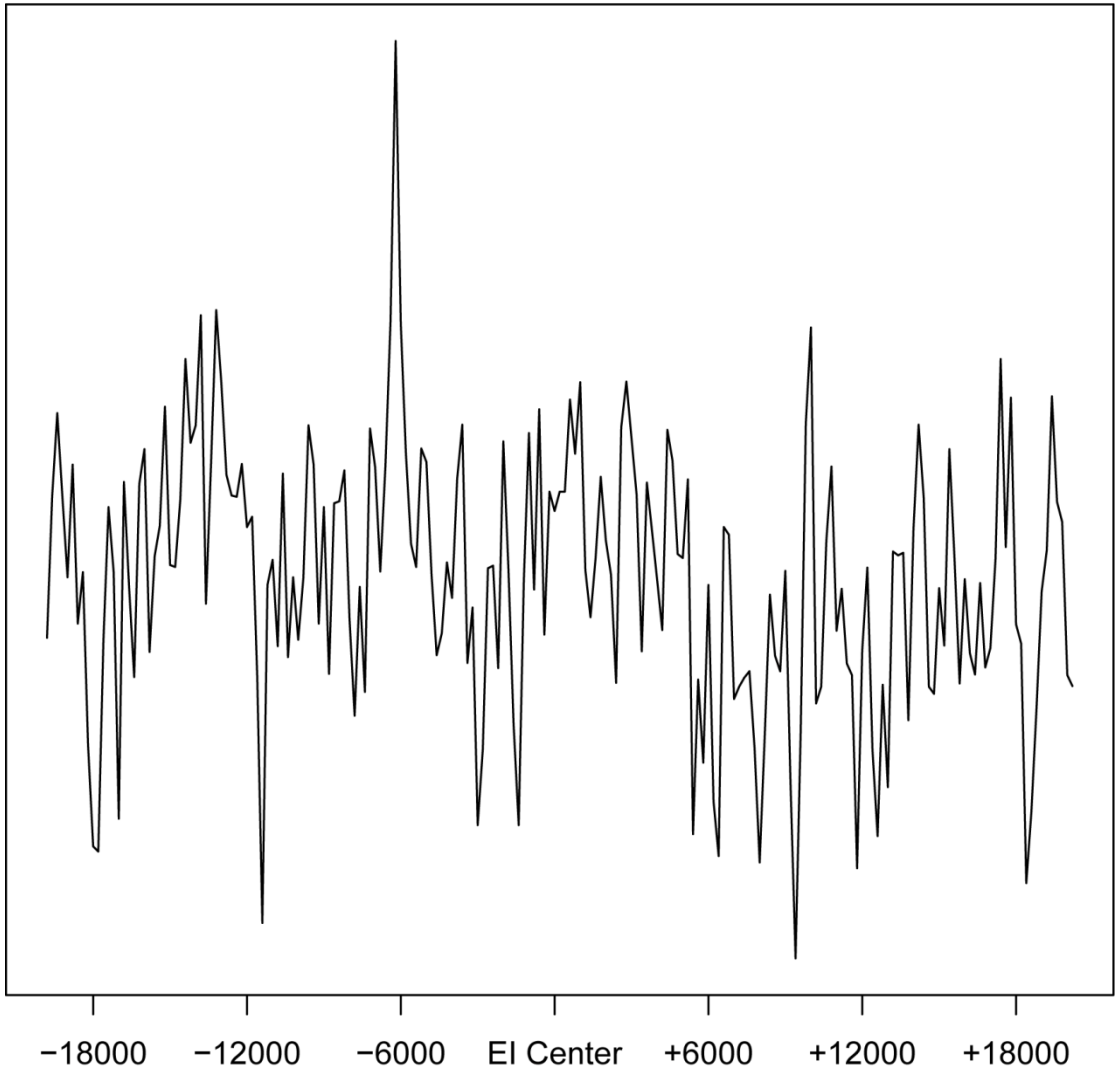

Supplement: Additional file 7 — Figure S5. Description: Nucleosome density in EIs and adjacent regions. We compared common EIs of HA, HAEC and HPF with nucleosome maps of GM12878 (Supplementary Table S3), to overcome potential lineage specificity among those cell types. [file 1471-2164-13-566-S7.pdf]

Fig. S6

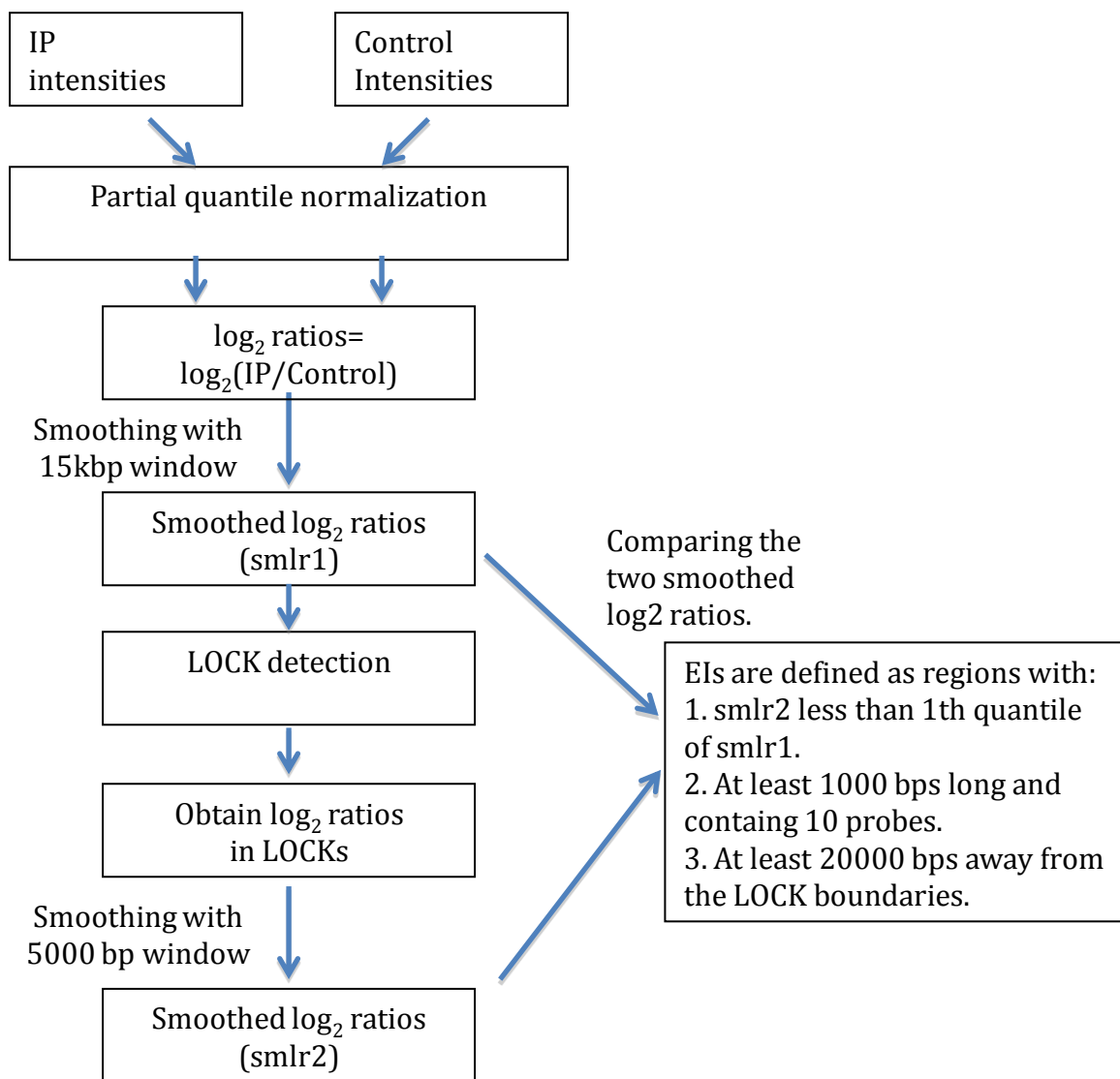

Supplement: Additional file 8 — Figure S6. Description: Flow diagram of EI detection. [file 1471-2164-13-566-S8.pdf]
