# Supplementary material for: Euchromatin islands in large heterochromatin domains are enriched for CTCF binding and differentially DNA-methylated regions
Source: BMC Genomics. 2012 Oct 26;13:566. doi: 10.1186/1471-2164-13-566 (PMC3507770; doi:10.1186/1471-2164-13-566)
Supplement: Additional file 9 — Table S3. Description: Public datasets used for analysis. [file 1471-2164-13-566-S9.docx]

**Supplementary Table S3**. Public datasets used for analysis

| Datasets | Cell Type | Resource |
| --- | --- | --- |
| ChIP-seq of CTCF, H3K4me3, H3K27me3, H3K36me3 and H3k9ac | HUVEC, H1 ES and NHLF | Ernst et al. Nature 2011 |
| DNase hyper sensitive sites | H1 ES and HUVEC | ENCODE/Duke-Dnase-seq |
| DNase hyper sensitive sites | NHLF | ENCODE/NW-Dnase-seq |
| RNA-seq | IMR90 | Hawkins et al, Cell Stem Cell, 2010 |
| Differentially methylated regions related to tissue specificity (T-DMRs) | Brain vs Liver vs Spleen | Irizarry et al., Nature Genetics, 2009 |
| Differentially methylated regions related to reprogramming (R-DMRs) | iPS cells vs fibroblasts | Doi et al., Nature Genetics, 2009 |
| Differentially methylated regions (DMRs) related to colon cancer (C-DMRs) | colon cancer vs normal | Irizarry et al., Nature Genetics, 2009; Hansen et al., Nature Genetics, 2011 |
| MNase-seq  Nucleosome Position | GM12878 | ENCODE/Stanford/BYU |
